# Identification of stably expressed reference small non‐coding RNAs for microRNA quantification in high‐grade serous ovarian carcinoma tissues

**DOI:** 10.1111/jcmm.12927

**Published:** 2016-07-15

**Authors:** Eliana Bignotti, Stefano Calza, Renata A. Tassi, Laura Zanotti, Elisabetta Bandiera, Enrico Sartori, Franco E. Odicino, Antonella Ravaggi, Paola Todeschini, Chiara Romani

**Affiliations:** ^1^ Division of Obstetrics and GynecologyASST Spedali Civili di BresciaBresciaItaly; ^2^ Department of Molecular and Translational MedicineUniversity of BresciaBresciaItaly; ^3^ Department of Medical Epidemiology and BiostatisticsKarolinska InstitutetStockholmSweden; ^4^ “Angelo Nocivelli” Institute of Molecular MedicineDivision of Obstetrics and GynecologyUniversity of BresciaBresciaItaly; ^5^ Division of Obstetrics and GynecologyUniversity of BresciaBresciaItaly; ^6^ Doctorate School of Molecular and Translational MedicineUniversity of MilanMilanItaly

**Keywords:** HGS‐OvCa, sncRNA, qPCR, endogenous reference, normalization

## Abstract

MicroRNAs (miRNAs) belong to a family of small non‐coding RNAs (sncRNAs) playing important roles in human carcinogenesis. Multiple investigations reported miRNAs aberrantly expressed in several cancers, including high‐grade serous ovarian carcinoma (HGS‐OvCa). Quantitative PCR is widely used in studies investigating miRNA expression and the identification of reliable endogenous controls is crucial for proper data normalization. In this study, we aimed to experimentally identify the most stable reference sncRNAs for normalization of miRNA qPCR expression data in HGS‐OvCa. Eleven putative reference sncRNAs for normalization (U6, SNORD48, miR‐92a‐3p, let‐7a‐5p, SNORD61, SNORD72, SNORD68, miR‐103a‐3p, miR‐423‐3p, miR‐191‐5p, miR‐16‐5p) were analysed on a total of 75 HGS‐OvCa and 30 normal tissues, using a highly specific qPCR. Both the normal tissues considered to initiate HGS‐OvCa malignant transformation, namely ovary and fallopian tube epithelia, were included in our study. Stability of candidate endogenous controls was evaluated using an equivalence test and validated by geNorm and NormFinder algorithms. Combining results from the three different statistical approaches, SNORD48 emerged as stably and equivalently expressed between malignant and normal tissues. Among malignant samples, considering groups based on residual tumour, miR‐191‐5p was identified as the most equivalent sncRNA. On the basis of our results, we support the use of SNORD48 as best reference sncRNA for relative quantification in miRNA expression studies between HGS‐OvCa and normal controls, including the first time both the normal tissues supposed to be HGS‐OvCa progenitors. In addition, we recommend miR‐191‐5p as best reference sncRNA in miRNA expression studies with prognostic intent on HGS‐OvCa tissues.

## Introduction

Epithelial ovarian cancer is the fifth most common type of cancer in women and the leading cause of mortality for gynaecological neoplasms, with high‐grade serous carcinoma (HGS‐OvCa) being the most frequent and aggressive histological type 1. The high mortality rate of HGS‐OvCa is because of the intrinsic biology of the disease that remains largely undetected, leading to a delayed diagnosis of patients presenting disseminated metastatic disease 2 and, consequently, a 5‐year survival rate of only 30% 3. Recent investigations report microRNAs (miRNAs) as important players in human carcinogenesis and aberrantly expressed in several cancers, including ovarian carcinoma tissues 4, 5. Those small (19–25 nucleotides) non‐coding transcripts act as gene regulators, modulating post‐transcriptional activity of multiple target mRNAs (oncogenes or tumour suppressor genes) by repression of translation or regulation of mRNA degradation after targeting the 3′ UTR 6, 7. Recently, several papers have identified HGS‐OvCa‐specific miRNA expression signatures as promising novel biomarkers associated with diagnosis, prognosis and response to therapy 8, 9. One of the most powerful and sensitive technique available for miRNA expression analysis is quantitative RT‐PCR, which commonly uses relative quantification as a strategy for data interpretation. In relative quantification, changes in miRNA expression in a given sample are expressed relative to another reference sample, after normalization using a stably expressed endogenous reference simultaneously determined. In this kind of study, similarly to mRNA expression analysis, the selection of valid and reliable endogenous normalizers is critical to minimize technical bias introduced at each step of miRNA retrotranscription and quantification and to avoid misinterpretation of results. Recently, it has been reported the recommendation to normalize target mRNA levels with reference genes belonging to the same RNA class 10. Consequently, for miRNA expression normalization, the endogenous control genes should belong to the small non‐coding RNA family (sncRNA), such as miRNA, small nuclear RNA (snRNA) and small nucleolar RNA (snoRNA).

Currently, a proper endogenous control‐based normalization strategy to be used in miRNA qPCR studies on cancer tissues has not been identified 11. Moreover, there is a lack of consensus in the literature on the most stably expressed endogenous controls that should be used in HG‐OvCa miRNA qPCR studies. Actually, most of the investigations report arbitrarily chosen endogenous controls, including miRNA, snRNA and snoRNA, without any experimental validation of their stability. In addition, the large majority of these studies report U6 (alias RNU6‐1) as an endogenous reference, although its use is still controversial, as a growing body of evidence demonstrate its high expression instability across normal and tumour tissues 12, 13.

To fill this gap of knowledge, the present investigation aims at identifying the most suitable sncRNAs as the endogenous controls for miRNA expression normalization in a wide and well‐characterized cohort of HGS‐OvCa tissues. Furthermore, as HGS‐OvCa histogenesis is still a matter of debate 14, 15, we aimed at validating the stability of the proposed endogenous control sncRNAs in both normal ovarian surface and fallopian tube epithelium, as normal tissues to be compared with the cancer counterpart.

## Materials and methods

### Selection of candidate reference sncRNAs

The candidate endogenous sncRNAs were chosen based on the literature suggesting their use for normalization of qPCR studies in HGS‐OvCa tissue samples, carrying out a Medline search using the MeSH terms ‘ovarian cancer’/‘ovarian carcinoma’ and ‘microRNA’/‘miRNA’ and ‘real‐time PCR’. In addition, endogenous miRNA recommended from Exiqon (Woburn, MA, USA), based on their stable and constitutive expression across different cells and tissues, were included in our study 11, 16, 17, 18.

### Patients cohorts

This study was performed following the Declaration of Helsinki set of principles and approved by the Research Review Board‐ the Ethic Committee‐ of the Spedali Civili, Brescia, Italy (study reference number: NP1676). Written informed consent was obtained from all patients enrolled. HGS‐OvCa tissue samples were obtained from 75 patients diagnosed and treated at the Division of Gynecologic Oncology of the University of Brescia (Italy), between 2003 and 2013. Normal control tissue samples were obtained from a total of 30 patients, undergoing surgery for benign pathologies (Table 1). Details are reported in Table S1.

**Table 1:** Clinic‐pathological characteristics of 75 HGS‐OvCa and 25 normal control patients

Characteristics	HGS‐OvCa	Normal control	
		Ovary	Tube
*n*	75	15	15
Age at diagnosis (mean years, range)	60 (36–84)	53 (49–62)	49 (42–58)
FIGO stage (%)			
III	53 (71)		
IV	22 (29)		
RT (%)			
0	18 (24)		
>0	57 (76)		

RT: residual tumour.

### RNA isolation, cDNA synthesis and quantitative real‐time PCR

Total RNA was extracted from tissue samples, homogenized with TissueLyser System (Qiagen), using TRIZOL reagent (Life Technologies, Carlsbad, CA, USA) and further purified using RNeasy MiniElute Cleanup kit (Qiagen, Germantown, MD, USA) with a modified protocol for co‐purification of small RNAs according to the manufacturer's instructions. RNA concentration and integrity was assessed as previously described 19. The miRCURY locked‐nucleic‐acid (LNA) Universal RT miRNA PCR system (Exiqon) based on universal reverse transcription followed by real‐time PCR amplification with sncRNA‐specific primers, was used for first‐strand cDNA synthesis and SYBR Green‐based amplification. Details are described in Table S2.

### Statistical analysis

Stability of candidate endogenous miRNA was evaluated using two different software programs commonly used in the experimental validation of reference genes, NormFinder 20 and geNorm 10. Both algorithms identify the most stable control gene in a given set of tissue samples and determine the optimal number of references required for reliable normalization of qPCR data.

Differences in sncRNA expression between groups were tested using linear models on log‐transformed sncRNA expression values, with *P* values and confidence intervals (CIs) estimation based on ‘White‐Huber’ heteroscedasticity corrected covariance matrices 21. To account for the presence of potential outliers, we fitted weighted least squares with weights computed by M‐estimation 22. To test non‐difference of sncRNA expression among groups, we used the two one‐sided test (TOST) approach, a type of intersection union test 23. Briefly an ‘equivalence range’ [ε_L_,ε_U_] is defined. The null hypothesis is set up so that if the 90% CI for the parameter of interest (*e.g*. the difference among group means) falls completely within the equivalence range, the null hypothesis can be rejected. Two one‐sided tests were conducted for both boundaries of the range. The overall null hypothesis is rejected at level α if the associated *P*‐value for each of the individual hypotheses is less than α (α = 0.05 in our analysis). Prior to conducting an equivalent test, we must define an equivalence range, in which we can consider the parameter of interest in the two groups to be substantively equal. While no fixed objective rules exist to guide the choice of the equivalence range because such choice may depend on substantive considerations, Wellek 24 suggested a strict tolerance value for a two sample *t*‐test of ±0.36 on log scale, and we decided to adopt this latest criteria in our data analysis.

Statistical analysis were performed with R 25 with additional package *robustbase* 26.

## Results

### Identification of reference sncRNAs

According to our Medline search criteria, we found 114 papers published from November 2007 to August 2015. Within these reports, we removed from analysis papers evaluating gene expression and genotyping studies, as well as miRNA expression studies performed on cell cultures, formalin‐fixed paraffin‐embedded tissues or plasma/serum/urines. Additional articles were excluded because full text was not available in the English language. The remaining 29 papers focused on miRNA expression evaluation in fresh frozen ovarian tumour tissues by real‐time PCR, using the following sncRNAs for data normalization, alone or in combination of two or three: U6 (25 times), SNORD48 (two times), miR‐92a‐3p (one time), let‐7a‐5p (one time), SNORD61 (one time), SNORD72 (one time), SNORD68 (one time). This panel of seven potential endogenous controls derived from the literature was integrated with four additional reference miRNAs, miR‐103a‐3p, miR‐423‐3p, miR‐191‐5p and miR‐16‐5p, selected and validated from Exiqon for normalization of miRNA expression levels in human tissue samples during real‐time PCR analysis (Table S1).

### Expression of candidate reference sncRNAs

**Figure 1:**
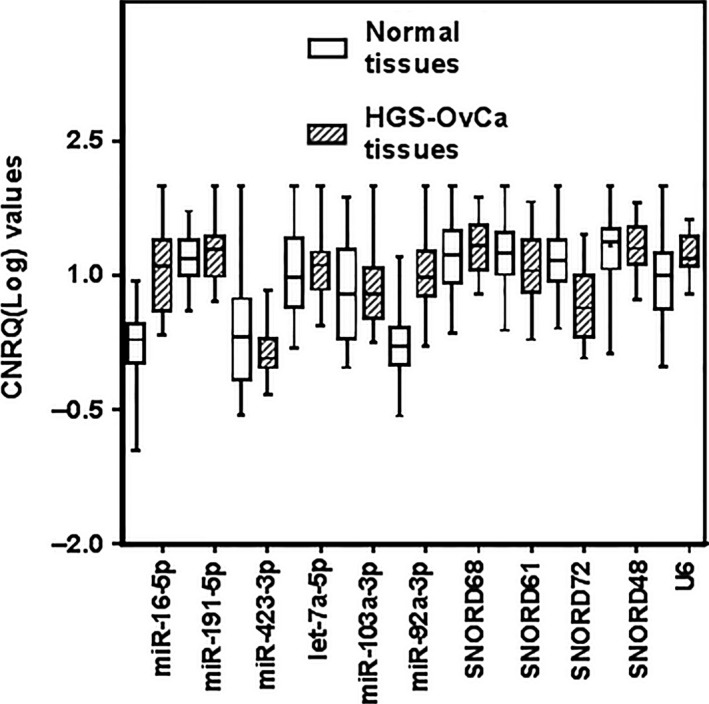
Expression levels of candidate reference sncRNAs in HGS‐OvCa (hatched boxes) and normal (open boxes) tissues. Values are given as calibrated normalized relative quantities (CNRQ). Boxes indicate IQR for the data of unmatched samples in each group. Error bars represent range of values.

Quantitative real‐time PCR was performed on 75 HGS‐OvCa, 15 normal luminal fallopian tube and 10 normal ovarian surface epithelia samples (representative of 15 patients) to assess the expression pattern of the 11 selected reference sncRNAs. All RNA samples isolated from malignant and normal specimens met the criteria of purity and integrity defined by A260/280 ratio (mean ± S.D., 2 ± 0.06) and RIN values (RIN ≥ 8). All RNA samples were verified to be free of any DNA contamination, by analysis of minus‐reverse transcriptase (‘‐RT’) controls in real‐time RT‐PCR experiments (Table S2). For the quantitative comparison of investigated candidate reference sncRNAs, the raw Cq values were converted to CNRQ, a normalization procedure which removes variations related to different cDNA starting quantities and correct for run‐to‐run variation using an internal control sample 27, and log‐transformed before statistical analysis. All candidate reference sncRNAs were expressed in abundance both in normal and in cancer tissues, with substantial higher spread in malignant samples compared with non‐malignant ones (Fig. 1). The sncRNAs showing the higher interquartile‐range (IQR) was miR‐103a‐3p (IQR = 2.05), while miR‐191‐5p showed the smallest (IQR = 0.92). Melting curve analysis confirmed the specificity of the PCR products, for each primer set tested. No amplification was detectable for each ‘no template control’ sample included in each assay run for each sncRNA primer set.

Applying the TOST method equates at computing (1‐2α)% CI (90% when α = 0.05) for the contrast of interest (Δ = difference between group means) and comparing it to the [ε_L_,ε_U_] range, if the CI is fully included in the equivalence range the null hypothesis of non‐equivalence can be rejected. Table 2 shows 90% CIs for all the contrasts of interest and the *P* values as well as an indicator of null hypothesis rejection based on |ε| = 0.36. Accordingly, considering the difference in sncRNA expression between malignant and non‐malignant samples, all sncRNA were substantially varying but SNORD48, whose ratio of the two groups averages (−0.282,+0.317) falls within the fixed equivalence range. Moreover, we performed the same analysis of statistical equivalence considering only the tumour sample cohort, grouped based on residual tumour (RT). Among malignant samples considering groups based on RT (RT = 0 *versus* RT > 0), miR‐191‐5p emerged as the most equivalent sncRNA (−0.232,+0.358).

**Table 2:** Log‐fold changes in reference sncRNA expression between HGS‐OvCa and normal control samples, and among non‐residual tumour and residual tumour (90% confidence intervals; *P* values for linear models; * null hypothesis of non‐equivalence rejected; [ε_L_,ε_U_] =[−0.36,0.36])

sncRNA	HGS‐OvCa *versus* normal control			RT = 0 *versus* RT > 0		
	Log FC	90% CI	*P*‐value	Log FC	90% CI	*P*‐value
miR‐16‐5p	−1.87	−2.36;−1.38	<0.001	−0.16	−0.43;0.11	0.328
miR‐191‐5p	−0.10	−0.44;0.24	0.625	0.06	−0.23;0.36*	0.725
miR‐423‐3p	0.21	−0.18;0.59	0.375	0.60	0.04;1.15	0.087
let‐7a‐5p	−0.20	−0.59;0.19	0.401	0.48	−0.01;0.98	0.108
miR‐103a‐3p	−0.15	−0.60;0.31	0.601	0.28	−0.35;0.90	0.465
miR‐92a‐3p	−1.89	−2.23;−1.55	<0.001	0.40	0.005;0.80	0.096
SNORD68	−0.23	−0.55;0.09	0.229	0.28	−0.14;0.71	0.274
SNORD61	0.33	−0.08;0.74	0.190	−0.05	−0.42;0.32	0.828
SNORD72	1.21	0.80;1.63	<0.001	0.25	−0.25;0.74	0.408
SNORD48	0.02	−0.28;0.32*	0.924	0.25	−0.14;0.64	0.287
U6	−0.53	−0.84;−0.23	0.003	0.56	0.06;1.06	0.066

### Analysis of candidate reference sncRNAs expression stability

**Figure 2:**
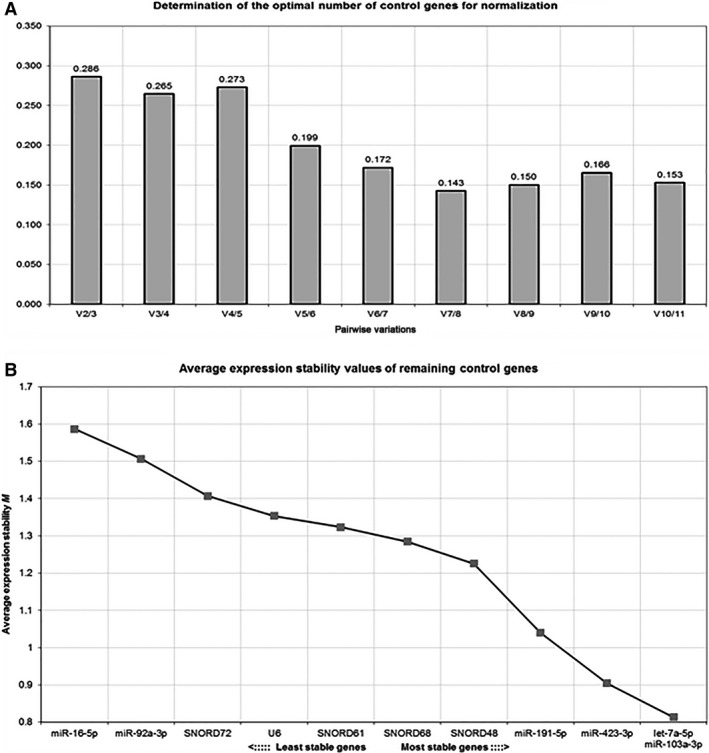
geNorm output charts. (**A**) Average expression stability value (M) of reference sncRNAs calculated at each step during stepwise exclusion of the least stable expressed reference. Starting from the least stable at the left, the sncRNA are ranked according to increasing expression stability, ending with the two most stable sncRNA on the right. (**B**) Normalization factor based on the pairwise variation (V), representing the levels of variation in average reference sncRNA stability with the sequential addition of each reference to the equation.

GeNorm and NormFinder software programs were used to compare and rank the 11 potential reference sncRNAs on the basis of their stability across tumour and normal tissue samples. According to the gene stability M value provided by geNorm, candidate reference sncRNAs were ranked from the least stable (highest M value) to the most stable (lowest M value). As an M value of 1.5 was defined as the upper limit for candidate reference, and 0.5 was the M value typically observed for stably expressed reference genes in homogeneous sample groups, values between 0.5 and 1 were considered as a characteristic when evaluating endogenous controls on heterogeneous samples like cancer and normal tissues 27. As shown in Figure 2A, all the studied sncRNAs achieved an overall medium to low expression stability, with M values ranging from 0.8 for let‐7a‐5p and miR‐103a‐3p to 1.6 for miR‐16‐5p (average geNorm M ≥ 1.2). Accordingly, let‐7a‐5p, miR‐103a‐3p and miR423‐3p were identified as the most stable sncRNAs in our panel of 75 HGS‐OvCa and 30 normal tissues, followed by miR‐191‐5p and SNORD48. In addition to the generated M value, geNorm software program calculates the optimal number of sncRNAs required for a reliable normalization of qPCR data, based on the variable V as the pairwise variation between sequential normalization factors. Taking 0.15 as the cut‐off value below which the inclusion of an additional reference gene is not required 10, the optimal number of reference sncRNAs was seven (Fig. 2B). It must be pointed out that the proposed 0.15 value should not be taken as a strict cut‐off, but rather as a guidance for the calculation of the optimal number of references. In our experimental system, the observed trend of changing V values when using additional sncRNAs reveals that the highest decrease in variation was achieved with the five most stable sncRNAs, in line with previous studies suggesting that a proper normalization strategy for relative gene quantification should include at least three to five endogenous references 10. The results generated from geNorm were compared and validated using NormFinder software program, whose algorithm rank the set of candidate normalization genes according to their expression stability. NormFinder identified SNORD48 as the best endogenous reference sncRNA, strictly followed by let‐7a‐5p and miR‐191‐5p, consistent with geNorm analysis which ranked let‐7a‐5p, miR‐191‐5p and SNORD48 among the fifth most stable sncRNAs (Table 3). Notably, U6, the reference sncRNA most frequently used as normalizer, was ranked poorly in both analysis. Both geNorm and NormFinder analysis were also in considerably closer agreement in the identification of miR‐16‐5p, miR‐92a‐3p and SNORD72 as the least stable sncRNAs in our cohort of samples.

**Table 3:** Candidate sncRNAs reference listed by their expression stability according to the NormFinder software program

Ranking order	sncRNA name	Stability value
1	SNORD48	0.393
2	let‐7a‐5p	0.395
3	miR‐191‐5p	0.422
4	SNORD68	0.426
5	U6	0.488
6	SNORD61	0.510
7	miR‐423‐3p	0.564
8	miR‐103a‐3p	0.565
9	SNORD72	0.688
10	miR‐92a‐3p	1.025
11	miR‐16‐5p	1.142

### *In silico* validation of candidate reference sncRNAs using RNA‐Seq data

To validate the stability of our candidate invariant sncRNAs, we performed the same analysis of statistical equivalence on RNA‐Seq data, based upon level 3 data generated by the TCGA research network (http://cancergenome.nih.gov/). RNA‐Seq data regarding 292 stage III‐IV HGS‐OvCa snap‐frozen tissues were available for analysis, with similar clinic‐pathologic characteristics compared with our cohort of samples. Among malignant samples, considering groups based on RT (RT = 0 *versus* RT > 0), miR‐191‐5p was confirmed to be the most equivalent sncRNA (−0.034,+0.231), as shown in Table 4.

**Table 4:** Statistical equivalence analysis of candidate reference sncRNA expression among non‐residual tumour and residual tumour HGS‐OvCa tissues, quantified using RNA‐Seq technology

sncRNA	HGS‐OvCa RT = 0 *versus* RT > 0		
	Estimate	90% CI	Width CI
miR‐16‐5p	0.0623	−0.14;0.27	−0.21
miR‐191‐5p	0.0989	−0.03;0.23	−0.13
miR‐423‐3p	0.1295	0.02;0.24	−0.11
let‐7a‐5p	0.0828	−0.17;0.33	−0.25
miR‐103a‐3p	0.1493	−0.09;0.38	−0.24
miR‐92a‐3p	0.1696	−0.02;0.35	−0.19
SNORD68	0.0635	−0.08;0.21	−0.14
SNORD61	−1.7428	−2.83;−0.65	−1.09
SNORD72	−1.2144	−2.28;−0.14	−1.06
SNORD48	0.0817	−0.08;0.25	−0.17
U6	0.1678	−0.004;0.38	−0.22

Conversely, only six normal fallopian tube and four normal ovary RNA‐Seq data were present in the TCGA database and no information about sample collection (*i.e*. macrodissected snap‐frozen biopsies or epithelial brushings) and tissue composition (*i.e*. epithelial purity) was provided. Due to this lack of information, we were prevented to perform the analysis of statistical equivalence on healthy tissue RNA‐Seq data.

## Discussion

Quantitative PCR is generally accepted as gold standard for miRNA measurement, and according to the guidelines for quality control and standardization of qPCR experiments, selection of reliable endogenous normalizer is a critical aspect to be considered for interpretation of data. Similarly to mRNA expression analysis, the choice of reference genes for miRNA qPCR data normalization has a great impact on the study outcome, as different normalization strategies can lead to different interpretation of data resulting in ambiguous biological conclusions 28.

As already postulated, miRNA may act ‘in cascade’ over several mRNA genes, regulating multiple target within the same pathway, thus small changes in miRNA expression could have important consequences for a given cellular function 29. Accordingly, the validation of endogenous normalizers is even more critical for miRNA qPCR experiments, considering that relatively small differences in miRNA expression may be biologically and clinically significant.

To date, no consensus strategy has been reached on the optimal normalization for miRNA expression studies, and irrespective of the guidelines suggesting a validation screening test on a subset of samples under analysis, endogenous references continue to be arbitrarily chosen without any experimental evidence. To our knowledge, no validated endogenous references have been identified for normalization of miRNA expression data in ovarian cancer tissues. Confirming this report, our comprehensive MEDLINE search of miRNA expression studies published between 2007 and the middle of 2015 showed that there was no uniform opinion on which reference should be used for reliable normalization in HGS‐OvCa samples. On the basis of the literature review, a total of seven sncRNAs were identified as putative references for miRNA expression studies in ovarian cancer tissues. Almost all of the published studies (25 out of 29) reported U6 as the reference of choice, while other sncRNAs, such as SNORD48, miR‐92a‐3p, let‐7a‐5p, SNORD61, SNORD72 and SNORD68, have been evaluated alone or in combination with U6 in the remaining papers. To increase the number of potential endogenous sncRNAs to be evaluated in our cohort of 75 HGS‐OvCa and 30 normal tissues, we decided to adopt a combination strategy integrating data from the literature with the following panel of reference sncRNAs: miR‐103a‐3p, miR‐423‐3p, miR‐191‐5p and miR‐16‐5p. They have been reported to be constitutively expressed in a variety of normal and pathological tissues, and validated from Exiqon for normalization of miRNA expression levels during real‐time PCR analysis. None of these potential endogenous controls had been previously evaluated in ovarian tissue samples. The particular design of our study has been characterized by several features, including: (*i*) wide cohort of snap‐frozen tumour tissue samples of HGS histological type, the most frequent and aggressive ovarian cancer, belonging to a single institution, (*ii*) inclusion of both ovarian surface and fallopian tube epithelia, as source of normal control tissues, (*iii*) stringent quality control of isolated total RNA, (*iv*) careful selection of putative reference sncRNAs, combining a Medline search result and validated endogenous controls from Exiqon, (*v*) use of optimized Exiqon primer sets with LNA technology, maximizing sensitivity and specificity in detecting sncRNA amplicons, (*vi*) qPCR performed with SYBR Green technology, that is cost‐effective and easy to apply in every laboratory setting, (*vii*) use of an inter‐run calibration sample, that showed an optimal correlation in sncRNA expression among all plates and (*viii*) experiments performed in triplicate for every sncRNA and every sample.

As known, reference genes are characterized by a stable expression across various samples and their use as normalizer in gene expression studies can correct for experimental variations related to sampling procedures, RNA extraction and RT efficiency. Besides, reliable quantification of miRNA expression levels faces some methodological problems, mainly related to the small size of mature miRNAs, their relatively low abundance in human tissues and the high degree of homology between miRNA family members 30. Accordingly, the choice of an appropriate detection system is an essential starting point to obtain accurate results. One of the strengths of our experimental method is the application of the Exiqon technology to RNA samples, that quantify miRNA expression in a two‐step PCR process of modified RT‐PCR, followed by a qPCR. In particular, starting from total RNA, a universal transcription system provided template for all mature miRNAs, overcoming the need for miRNA‐specific reverse transcription whose efficiency can vary among different miRNAs. Both PCR amplification primers were miRNA‐specific and chemically modified in the ribose moiety of nucleotides to stabilize the conformation of the sugar groups 31, according to the Exiqon technology. The conformation of LNA oligos resulted in enhanced hybridization properties and increased sensitivity and specificity in detection of scnRNAs. Moreover, the RT mechanism allowed the amplification of mature miRNA only, without amplification of pre‐miRNA whose interaction with oligos is prevented by the presence of the loop.

The aforementioned exceptional sensitivity, specificity and accuracy of the Exiqon qRT‐PCR system have recently been confirmed by Mestdagh *et al*. in the widest peer‐reviewed investigation of miRNA profiling platforms performed to date 31.

The second strength of our investigation relies on the use of both the normal tissues suggested to be the initiating points of the HGS‐OvCa carcinogenetic process. Indeed, there has been increasing evidence that HGS‐OvCa might originate in oviductal fimbriae and metastasize to the ovary 14, but the alternative hypothesis assuming that ovarian carcinomas may originate within ovarian stroma in inclusion cysts lined by ovarian surface epithelium is still under consideration 32, 33. Given these assumptions, our choice to include in the present experimental setting both the putative normal controls for HGS‐OvCa represents an innovative approach, as ovarian and fallopian tube epithelia have been poorly investigated together as normal controls in previously reported HGS‐OvCa gene expression studies. Moreover, our approach represents an important point to obtain reliable expression data on reference and eventually target sncRNAs, comparing HGS‐OvCa and its dually suggested normal counterpart.

To determine the best performing reference sncRNAs, we analysed our results with geNorm and NormFinder software programs, two algorithms commonly used in gene expression studies and specifically developed for reference gene evaluation and selection 10, 20. While geNorm indicated let‐7a‐5p, miR‐103a‐3p, miR‐423‐3p, miR‐191‐5p and SNORD48 as the four most stably expressed reference sncRNAs, NormFinder identified SNORD48, strictly followed by let‐7a‐5p and miR‐191‐5p, as the three sncRNAs with the best stability. However, the previously performed statistical analysis on miRNA expression of our cohort of samples revealed that only SNORD48 and miR‐191‐5p showed a significant equivalent expression in malignant *versus* non‐malignant ovarian tissues or among malignant samples grouped according to RT respectively. The same powerful parametric approach has been recently reported by our group, as a reliable tool to identify optimal reference genes for gene expression normalization in endometrial cancer tissues 19. Let‐7a‐5p, although highly ranked by NormFinder and indicated among the most stable reference genes in geNorm analysis, did not fulfil the strict criteria of equivalency established by our statistical approach. In this context, it is not advisable to blindly accept the best combination suggested by geNorm and NormFinder, as both algorithms included sncRNAs showing differences in expression level between normal and ovarian tumour tissues. Actually, combining the results from the powerful statistical analysis and the expression stability performed on the expression level of 11 candidate reference sncRNAs, SNORD48 consistently emerged as the most stably expressed sncRNA, regardless of sample type, to be used as normalizer for relative miRNA quantification in HGS‐OvCa samples *versus* normal controls. Notably, SNORD48 was already reported among the most stably expressed sncRNAs in endometrial cancer and renal cell carcinoma tissues 13, 34. Conversely, U6, the most commonly reported normalization sncRNAs for miRNA expression studies, did not fulfil the criteria of constant expression between our cohorts of normal and tumour samples, as already described for other malignancies 11, 12, 17.

The second reference sncRNA resulted from our statistical analysis, confirmed to be equivalent within the group of tumour samples regardless of RT, was miR‐191‐5p. It is noteworthy that RT is considered the most informative prognostic factor in advanced‐stage HGS‐OvCa 35. Given this assumption, miR‐191‐5p should be recommended as reference sncRNA in miRNA expression studies with prognostic intent of HGS‐OvCa tissues. Moreover, miR‐191‐5p equivalent expression among HGS‐OvCa tissues was successfully validated by a further *in silico* analysis on RNA‐Seq data, available at TCGA. This additional validation strengthens our results, as RNA‐Seq technology provides a precise and accurate quantification of transcript levels.

To the best of our knowledge, the current investigation reports the first evaluation of a panel of putative reference sncRNAs in HGS‐OvCa tissue samples, the most frequent ovarian carcinoma histotype, using either commonly reported software programs for gene expression normalization or a novel powerful statistical approach. Taking our findings together, the present study proposes, for the first time, SNORD48 as the best reference sncRNA for relative quantification in miRNA expression studies comparing HGS‐OvCa tissues and normal controls. Importantly, this is also the first study evaluating and validating the equivalency and the stability of SNORD48 expression, other than in tumour samples, in both the normal tissues considered to initiate the malignant transformation. Finally, this is the first report supporting the use of miR‐191‐5p in HGS‐OvCa tissues with different RT, in agreement with results provided by Exiqon regarding other solid malignancies.

## Funding

CARIPLO Foundation grant number 2013‐0815 to E. Sartori.

## Conflict of interest

The authors confirm that there are no conflicts of interest.

## Author contribution

All authors confirmed that they have contributed to the intellectual content of this paper and have met the following three requirements: (*i*) Substantial contributions to the conception and design, acquisition of data, or analysis and interpretation of data; (*ii*) Drafting or revising the article for intellectual content; and (*iii*) Final approval of the published article.

## Supporting information


**Table S1** Characteristics of candidate sncRNAs selected for evaluation of expression stability.

**Table S2** Median and range of Cqs with and without reverse transcriptase for each sncRNA analysed.
