# Supplementary material for: Identification of stably expressed reference small non‐coding RNAs for microRNA quantification in high‐grade serous ovarian carcinoma tissues
Source: J Cell Mol Med. 2016 Jul 15;20(12):2341–8. doi: 10.1111/jcmm.12927 (PMC5134371; doi:10.1111/jcmm.12927)
Supplement: Supplementary file 1 — Table S1 Characteristics of candidate sncRNAs selected for evaluation of expression stability. [file JCMM-20-2341-s001.docx]

**Supporting Table 1**

**Characteristics of candidate sncRNAs selected for evaluation of expression stability**

| **sncRNA** | **HGNC**  **symbol** | **Seq. accession number** | **Target sequence lenght (nt)** |
| --- | --- | --- | --- |
| SNORD48 | SNORD48 | NR_002745 | 63 |
| SNORD72 | SNORD72 | NR_002583 | 80 |
| SNORD61 | SNORD61 | NR_002735 | 73 |
| SNORD68 | SNORD68 | NR_002450 | 72 |
| U6 | RNU6-1 | NR_004394 | 106 |
| miR-16-5p | MIR16-1 | NR_029486 | 22 |
| miR-191-5p | MIR191 | NR_029690 | 23 |
| miR-423-3p | MIR423 | NR_029945 | 23 |
| let-7a-5p | MIRLET7A | NR_029476 | 22 |
| miR-103a-3p | MIR103A2 | NR_029519 | 23 |
| miR-92a-3p | MIR92A2 | NR_029509 | 22 |
