# Supplementary material for: Identification of stably expressed reference small non‐coding RNAs for microRNA quantification in high‐grade serous ovarian carcinoma tissues
Source: J Cell Mol Med. 2016 Jul 15;20(12):2341–8. doi: 10.1111/jcmm.12927 (PMC5134371; doi:10.1111/jcmm.12927)
Supplement: Supplementary file 2 — Table S2 Median and range of Cqs with and without reverse transcriptase for each sncRNA analysed. [file JCMM-20-2341-s002.docx]

**Supporting Table 2**

**Median and range of Cqs with and without reverse transcriptase for each sncRNA analyzed.**

| **sncRNA** | **RT+** | | **inter-assay variation coefficient** | **RT-** | |
| --- | --- | --- | --- | --- | --- |
|  | **median** | **range** |  | **median** | **range** |
| SNORD48 | 23.7 | 21.55-27.79 | 0.044 | n/a | n/a |
| SNORD72 | 28.77 | 25.66-32.08 | 0.05 | n/a | n/a |
| SNORD61 | 25.07 | 22.47-28.20 | 0.046 | n/a | n/a |
| SNORD68 | 22.23 | 19.72-25.19 | 0.053 | n/a | n/a |
| U6 | 22.59 | 19.86-26.61 | 0.06 | n/a | n/a |
| miR-16-5p | 22.43 | 16.97-26.84 | 0.073 | n/a | n/a |
| miR-191-5p | 26.52 | 23.96-28.65 | 0.037 | n/a | n/a |
| miR-423-3p | 25.3 | 19.20-27.72 | 0.064 | n/a | n/a |
| let-7a-5p | 21.61 | 18.32-24.39 | 0.066 | n/a | n/a |
| miR-103a-3p | 22.67 | 18.65-25.41 | 0.076 | n/a | n/a |
| miR-92a-3p | 25.79 | 20.25-28.82 | 0.062 | n/a | n/a |
